# Supplementary material for: Leucine Reconstitutes Phagocytosis-Induced Cell Death in E. coli-Infected Neonatal Monocytes—Effects on Energy Metabolism and mTOR Signaling
Source: Int J Mol Sci. 2021 Apr 20;22(8):4271. doi: 10.3390/ijms22084271 (PMC8074332; doi:10.3390/ijms22084271)
Supplement: Supplementary file 1 [file ijms-22-04271-s001.zip › ijms-1138597-supplementary.pdf]

Supplementary material to

"Leucine induces phagocytosis induced cell death (PICD) in cord blood monocytes by uncoupling glycolysis from mTOR signaling" by Dreschers et al.

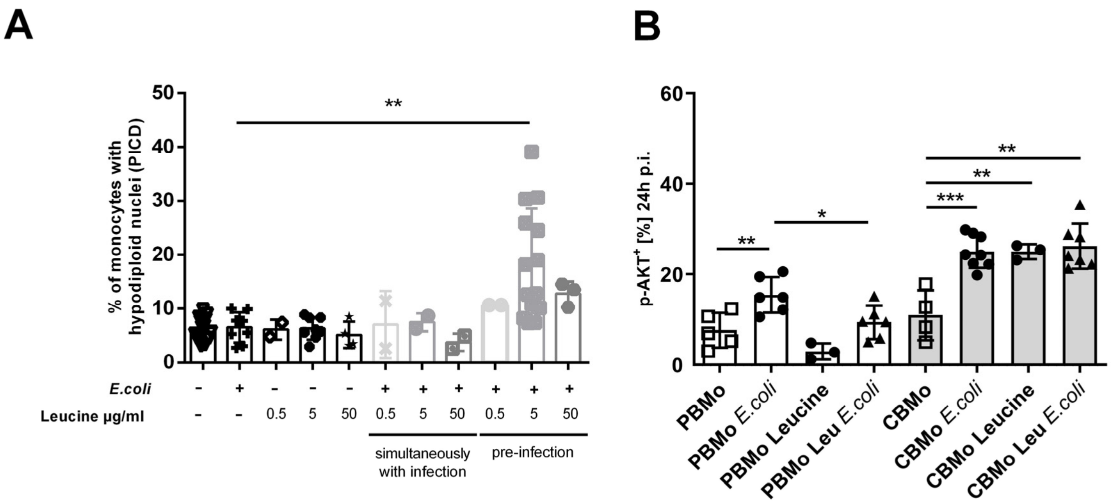

Supplementary Figure S1: Enhanced PICD in CBMo after leucine pre-treatment and AKT phosphorylation. PICD was detected in CBMo with indicated treatment (Leu, leucine) for the infection interval of 24 h (A). The MOI was 50 (**, p<0.01). Chart (B) shows Mo exhibiting phosphorylated AKT under indicated conditions (all bars in charts show SD; Student`s t-test *, p<0.05; **, p < 0.01; ***, p < 0.005).

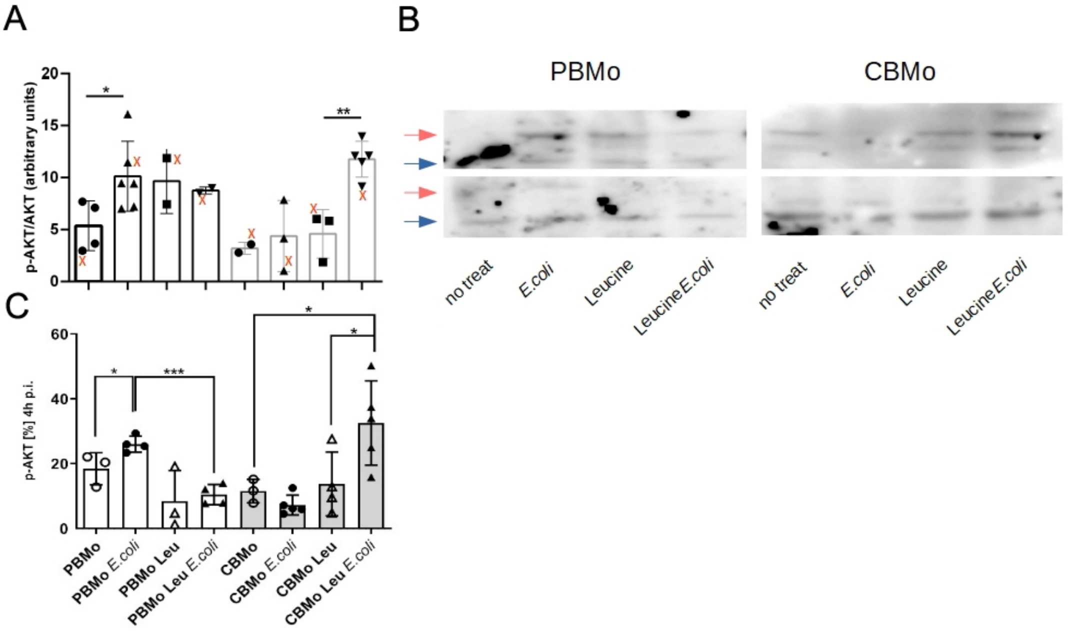

Supplementary Figure S2: Comparison of FACS analysis with immunoblot technique. Lysates of Mo treated as given in the legend were stained with AKT (blue arrows) and phospho-AKT (red arrows) on immunoblot example in (B). Three independent experiments were quantified via image j (A, red crosses designate the data set presented in the immunoblot to the right) and for comparison, the FACS analysis (C). All bars in charts show SD; lines, Student`s t-test *, p<0.05; **, p < 0.01; ***, p < 0.005).

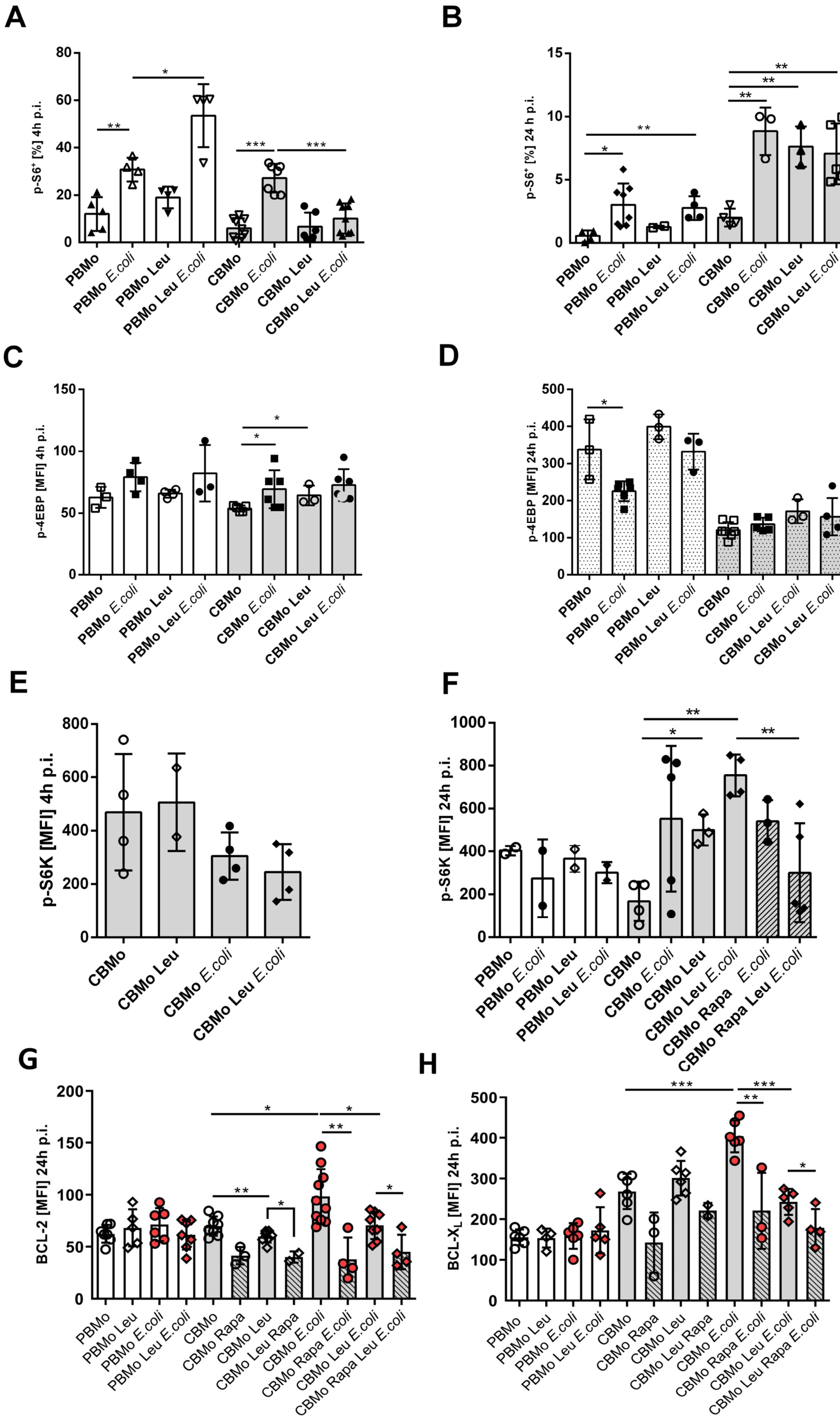
A
p-S6+ [%] 4h p.i.
0
20
40
60
80
PBMo
PBMo E.coli
PBMo Leu
PBMo Leu E.coli
CBMo
CBMo E.coli
CBMo Leu
CBMo Leu E.coli
B
p-S6+ [%] 24 h p.i.
0
5
10
15
PBMo
PBMo E.coli
PBMo Leu
PBMo Leu E.coli
CBMo
CBMo E.coli
CBMo Leu
CBMo Leu E.coli
C
p-4EBP [MFI] 4h p.i.
0
50
100
150
PBMo
PBMo E.coli
PBMo Leu
PBMo Leu E.coli
CBMo
CBMo E.coli
CBMo Leu
CBMo Leu E.coli
D
p-4EBP [MFI] 24h p.i.
0
100
200
300
400
500
PBMo
PBMo E.coli
PBMo Leu
PBMo Leu E.coli
CBMo
CBMo E.coli
CBMo Leu E.coli
CBMo Leu E.coli
E
p-S6K [MFI] 4h p.i.
0
200
400
600
800
CBMo
CBMo Leu
CBMo E.coli
CBMo Leu E.coli
F
p-S6K [MFI] 24h p.i.
0
200
400
600
800
1000
PBMo
PBMo E.coli
PBMo Leu
PBMo Leu E.coli
CBMo
CBMo E.coli
CBMo Leu
CBMo Leu E.coli
CBMo Rapa E.coli
CBMo Rapa Leu E.coli
G
BCL-2 [MFI] 24h p.i.
0
50
100
150
200
PBMo
PBMo Leu
PBMo E.coli
PBMo Leu E.coli
CBMo
CBMo Rapa
CBMo Leu
CBMo Leu Rapa
CBMo E.coli
CBMo Rapa E.coli
CBMo Leu E.coli
CBMo Rapa Leu E.coli
H
BCL-XL [MFI] 24h p.i.
0
100
200
300
400
500
PBMo
PBMo Leu
PBMo E.coli
PBMo Leu E.coli
CBMo
CBMo Rapa
CBMo Leu
CBMo Leu Rapa
CBMo E.coli
CBMo Rapa E.coli
CBMo Leu E.coli
CBMo Leu Rapa E.coli

Supplementary Figure S3: Comparison of FACS analysis of mTOR downstream targets. Percentage of phosphorylated ribosomal protein S6 (A, B), mean fluorescent intensity (MFI) of 4-EBP-1 (4-EBP, C, D) and MFI of phosphorylated S6 Kinase (p-S6K, E and F) was assessed. The anti-apoptotic factors BCL-2 (G) and BCL-X_L (H) were detected in Mo with indicated treatment (Leu, leucine; Rapa, rapamycin) for the indicated infection interval. The multiplicity of infection (MOI) was 50 (all bars in charts show SD; lines, Student`s t-test *, p<0.05; **, p < 0.01; ***, p < 0.005).
